# Progress in Nano-Engineered Anodic Aluminum Oxide Membrane Development

**DOI:** 10.3390/ma4030487

**Published:** 2011-02-25

**Authors:** Gerrard Eddy Jai Poinern, Nurshahidah Ali, Derek Fawcett

**Affiliations:** Murdoch Applied Nanotechnology Research Group, Faculty of Minerals and Energy, School of Engineering and Energy, Murdoch University, Murdoch 6150, Australia; E-Mails: N.Ali@murdoch.edu.au (N.A.); D.Fawcett@murdoch.edu.au (D.F.)

**Keywords:** anodic aluminum oxide, nanotechnology, nano-materials, nano-devices, tissue engineering

## Abstract

The anodization of aluminum is an electro-chemical process that changes the surface chemistry of the metal, via oxidation, to produce an anodic oxide layer. During this process a self organized, highly ordered array of cylindrical shaped pores can be produced with controllable pore diameters, periodicity and density distribution. This enables anodic aluminum oxide (AAO) membranes to be used as templates in a variety of nanotechnology applications without the need for expensive lithographical techniques. This review article is an overview of the current state of research on AAO membranes and the various applications of nanotechnology that use them in the manufacture of nano-materials and devices or incorporate them into specific applications such as biological/chemical sensors, nano-electronic devices, filter membranes and medical scaffolds for tissue engineering.

## 1. Introduction

The anodization process of metals has been used by industry to protect metal components from corrosion for approximately 90 years. During this electro-chemical process the surface chemistry of the metal is changed, via oxidation, to produce an anodic oxide layer that is thick enough to stifle further oxidation. Aluminum metal (Al), because of its high strength to weight ratio, has found numerous engineering applications [1,2] and as early as the mid 1920s components on seaplanes used in aviation transportation were being anodized in chromic acid [3]. Two types of anodic Al oxide exist; the first is a non-porous barrier layer that is thin, hard, wear resistant and behaves as an electrical insulator. The second, a thicker porous oxide structure, is called the anodic aluminum oxide (AAO) layer. This layer structure has a high aspect ratio and consists of a porous structure. In engineering applications, this pore structure must be sealed to prevent corrosion. Oxide layers generated during anodization can also be produced on materials such as: magnesium (Mg), niobium (Nb), silicon (Si), tantalum (Ta), tin (Sn), titanium (Ti), tungsten (W), zinc (Zn) and zirconium (Zr).

In recent years, there has been a renewed interest in AAO layers for use as templates in a variety of nanotechnology applications. This is due to the highly controllable pore diameter and cylindrical shape, their periodicity and their density distribution. Using the conventional anodization process the arrangement of the pores is quite disordered, however Masuda *et al*. [4] in 1998, using a two-step anodization process was able to produce a highly ordered hexagonal pore structure from a set of pre-arranged macroscopic parameters. These controllable macroscopic parameters dictated the resulting nano scaled structure that is formed in the AAO layer, thus producing a nano array that can be used in a variety of nanotechnology applications.

In the last decade, there has been a veritable explosion of ideas for the potential applications of nano-structured materials. To date, almost all traditional methods of producing synthetic materials have been revisited and investigated for possible use in the manufacture of nanomaterials. Nanomaterials are materials with basic structural units, grains, particles, tubes, spheres, fibers or other constituent components in the range of 0.1 nm to 100 nm. They are increasingly becoming the subject of many investigations in several fields such as materials science, biotechnology and biomedicine [5,6]. Nanomaterials can be made from a wide range of solid materials such as metals, ceramics, polymers, organic materials and composites. They can come in a wide range of morphologies namely spheres, rods, tubes and plates. In addition, these materials can be grown or self-assembled to replicate the dimensions of natural entities such as collagen fibers [7].

Due to their small size, these nanomaterials produce composite materials with novel properties and significant improvements in their functionality. Incorporation of nanomaterials into other bulk materials can also benefit the final product, e.g. carbon fibers in a polymer matrix [8]. One-dimensional materials such as rods, tubes and fibers can be manufactured by various chemical techniques, but the poly diversity of the final product is generally a problem. To resolve this problem researchers have used porous scaffolds, with controllable cavities that can be optimized, to manufacture regular and uniform rods or tubes. Anodic porous alumina has been used in the past to manufacture nanofiltration membranes, membrane reactors and even bioactive surfaces for tissue engineering (osteoblast and skin). In addition, techniques such as electro-deposition, polymerization, sol-gel and chemical vapor deposition (CVD) have used the regular porous alumina cavities to manufacture nanowires, nanotubes and quantum rods [9,10].

It will be the efficient and economical processes for creating materials with very low poly-diversity in the nano structures that will succeed. For example; CNTs can be produced using elaborate CVD techniques that incorporate extensive ultra high vacuum (UHV) instrumentation, which allows greater control over the final nanocarbon products. This technique gives good quality control of the final products but it is a very expensive process. On the other hand, a much more economical method of producing nanocarbons can be achieved by simply sparking two graphite rods, a plethora of nanostructures are generated without much control over the products being engineered. An alternative technique that provides good control over the final nanocarbon products and is economical advantageous uses an alumina template for the manufacture of nanostructures. This technique is highly reproducible and can be done safely at ambient conditions in the general laboratory.

## 2. Aluminum (III) Oxide, [Al_2_O_3_], Alumina

### 2.1. Introduction

Al metal is a substantial component of the Earth’s crust, (approximately 8%) but due to its reactivity with naturally occurring oxygen in the atmosphere, it is found in combination with other materials. It is a constituent of bauxite ore and it only became viable to process about 100 years ago. Following the pioneering work of Sir Humphrey Davy and physicist Hans Christian Oersted (who is credited with the manufacture of the first Al nodules), Karl Bayer was able to refine and improve the manufacturing of alumina from bauxite. Alumina (Al_2_O_3_) is the only known stable oxide of Al and contains 52.8% of the element by weight. Today, due to its wide applications, Al is the most produced non-ferrous metal [11].

### 2.2. Types of Oxide Films on Aluminum

Al is a reactive metal that reacts readily with the oxygen present in the atmosphere at ambient temperatures to create a thin amorphous nanometer (1–10 nm) oxide layer. The thickness of the oxide layer is temperature dependent and at temperatures above 500 °C, both amorphous and crystalline aluminas are present [12]. This layer has the advantage of preventing further dissolution of the Al and thus provides an effective protective barrier. This is in direct contrast to the permeable oxide layer that is built up on ferrous metal surfaces; thus allowing further corrosion to continue. The protective oxide layer on the surface of Al has enabled this metal to be used in a variety of industrial applications [13]. This oxide layer is very important and the control of its morphology and surface features are critical to many applications. It was shown as early as 1935 by Verwey [14] and other researchers [15] that that the anodic film will react further with the environment, resulting in hydration of the outer surface of the barrier film. Thus, the oxide structure consists of two distinct layers, a hydrated porous layer growing on top of a thin inert dense layer, normally called the barrier layer. The formation mechanisms of the barrier layer and the nano porous oxide layer are discussed further in Section 3.2.4.

## 3. Anodic Aluminum Membrane (AAO)

### 3.1. Introduction

A regular self-organized porous nanostructure can be formed when Al is anodized in certain acidic media. In Section 3 we discuss the mechanisms behind the growth of the porous nanostructure and the controllable macroscopic parameters; such as voltage, acid type and concentration that control the formation of the porous nanostructure [4,13]. This porous nanostructure is electrically insulating, optically transparent or semi-transparent, chemically stable, bio-inert and a biocompatible material. The nanostructure is well defined and has a highly ordered nano-architectured that enables these structures to be used as templates [9]. The creation of these templates is possible due to the well-developed and refined anodized techniques and electro-chemical parameters that are employed [10]. The technique and the electro-chemical parameters make it possible to control a number of surface parameters [13], for example the size of the pore diameter can be adjusted from 5 nm to 10 µm [16].

### 3.2. AAO Electrochemistry

**Figure 1:**
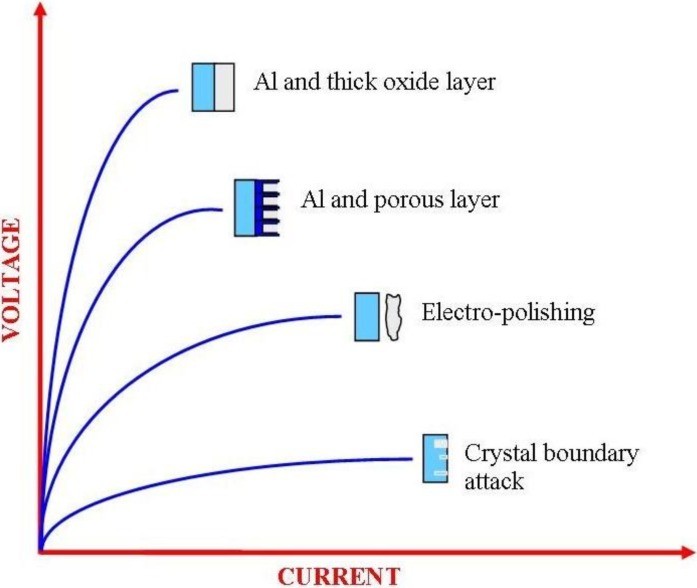
Anodic polarization of aluminum in different electrolyte solutions (reproduced from Reference [22]).

To use Al effectively for a wide variety of applications means that the metal needs to be protected. Anodization of Al was first used on an industrial scale in the mid 1920s, since then the range of applications has steadily increased and accordingly the anodization process has been actively investigated and refined [17]. It is known that during the anodization process, Al produces a highly impervious protective layer on its surface. The anodic layer parameters such as barrier layer thickness, pore diameter and pore height are directly dependent upon the steady-state voltage used in the creation of the layer [18,19,20,21]. This is graphically presented in Figure 1, which also shows the results of varying both the voltage and the current on the surface of an Al sample. It can be seen that at low voltages and high currents, pitting at the crystallographic boundaries begins, while at higher voltages and lower currents, electro-polishing effects take place. As the voltage is further increased, the current decreases and a porous layer will form. And finally, at extremely low currents and high voltages, a thick layer of Al oxide is formed.

#### 3.2.1. Materials

**Figure 2:**
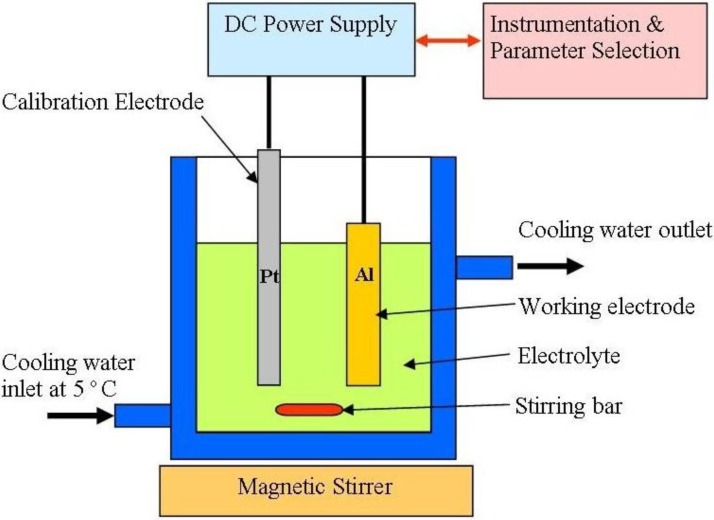
Experimental equipment used to produce anodized aluminum oxide.

The advantage of using porous alumina as a template is that it enables selected nano-pore fabrication using general laboratory equipment, see Figure 2. The proportionality of the cell size with respect to the electrochemical conditions allows one to macroscopically dictate the growth and final size of the pores manufactured. The equation is a simple one where an Al substrate is used with a particular acid to achieve nano-pores with an empirical derived set of electrochemical conditions.

**Al + acid + voltage = nano-pores**

Initially, the quality of the Al substrate, its surface structure and/or any surface pre-treatments will have a significant impact on the morphology and the resulting nano-structures formed on the substrate surface during the anodization process. To begin with, the Al substrate will have a pre-existing oxide layer over its surface, which is normally produced by the ambient oxygen in the atmosphere. In addition, the substrate could also have a pre-existing surface structure produced by a mechanical, thermal, chemical and electrochemical process. All of these surface treatments prior to anodization can have a significant impact on the self-ordering of the pore structures that form on the surface of the substrate during the anodization process. This is because the pore nucleation mechanism is a combination of both random nucleation and nucleation produced by the effects of surface defects, such as scratches, pits, impurities and grain boundaries. It should also be pointed out that during the anodization process, surface defects are favored sites for pore nucleation. Furthermore, studies have shown that the presence of alloying elements in the Al substrate not only tend to reduce the rate of growth of the forming oxide layer, but also influence the structure of the oxide layer during the anodization process [23].

**Figure 3:**
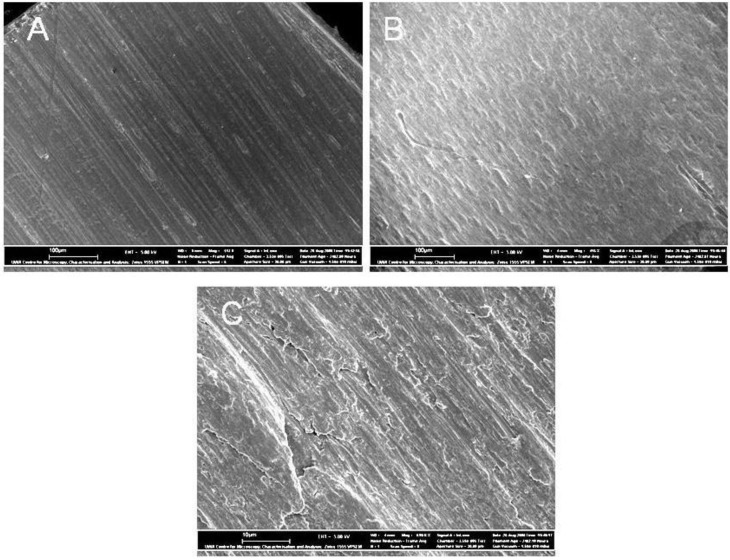
Field emission scanning electron microscopy images of annealed and non-annealed Al substrate. Annealing conditions: 500 °C for 5 hours. (**A**) Non-annealed Al, substrate, (**B**) Annealed Al substrate (scale bar = 100 µm), and (**C**) higher magnification of annealed substrate (scale bar = 10 µm).

The purity of the Al substrates used has a direct bearing on its dissolution rate in the acidic conditions of the electrolyte and thus, high purity Al is generally used. A purity of 99.99% is common and even 99.999% has been used. The use of a high purity foil strip allows the upscale of this technology for the large-scale manufacture of one dimensional nano-sized materials and components. A typical pre-treatment of an Al strip begins by first, degreasing the strip using acetone or a similar solvent, then electrochemically polished in a solution of 3 M NaOH for 5 minutes and finally washed in milli-Q water. This is followed by the annealing process; which is applied to the Al strip to reduce the mechanical stresses in the material and to modify the grain boundaries, since both these factors can have a significant effect on pore nucleation [24,25,26]. Annealing is usually performed at a temperature close to two-thirds of the melting point of Al (usually between 400 to 500 °C) in an inert atmosphere, such as nitrogen or argon [27]. The strip is kept at this temperature for a suitable soaking period (usually between 3 and 5 h) and then allowed to slowly cool in the inert atmosphere to prevent oxidation. During the annealing process the Al grain size increases and the surface of the strip becomes rougher and the larger grain boundaries can be seen, see the field emission scanning electron microscopy images in Figure 3. Further surface treatment may be needed, if not the strip is cleaned and stored for future use.

#### 3.2.2. Electrolytes

##### 3.2.2.1. Acids

A wide range of acids have been used in the anodization process involving Al. The range of acids has expanded with time and experience to address specific applications. In particular, nano-features demanded by a particular application will favor the use of one acid type over another and some researchers have used a combination of acids. Table 1 below presents a sample of the major acids used to produce porous oxide growth on an Al substrate. The acids listed in Table 1 are used in varying combinations depending upon the researcher’s experience. If the reader is interested, the exact parameter details can be found in the related reference. However, the majority of researchers tend to use oxalic, phosphoric and sulfuric acid to manufacture nano-pores ranging in size from around 10 to 240 nm (see Table 2).

**Table 1:** Major acid components of typical electrolyte types used to produce porous oxide layer on an aluminum substrate.

Main Acid used in Electrolyte	Molecular Formula	Concentration (M)	Pore Size Range (nm)	References
Acetic	CH_3_CO_2_H	1	Not specified	[28]
Citric	HO_2_CCH_2_(OH)(CO_2_H)CH_2_CO_2_H	0.1 to 2	90 to 250	[30,31,32]
Chromic	H_2_CrO_4_	0.3, 0.44	17 to 100	[33,34,35]
Glycolic	CH_2_(OH)CO_2_H	1.3	35	[30]
Malic	HO_2_CH_2_CH(OH)CO_2_H	0.15 to 0.3	Not specified	[30,32]
Malonic	CH_2_(CO_2_H)_2_	0.1 to 5	Not specified	[32,36,37]
Oxalic	C_2_H_2_O_4_	0.2 to 0.5	20 to 80	[40,41,42,43,44,45,46,47,48]
Phosphoric	H_3_PO_4_	0.04 to 1.1	30 to 235	[9,34,49,50]
Sulfuric	H_2_SO_4_	0.18 to 2.5	12 to 100	[41,48,51,52]
Tartaric	HO_2_CCH(OH)CH(OH)CO_2_H	0.1 to 3	Not specified	[30,32,37]

**Table 2:** Voltage and times of three commonly used electrolytes for the production of porous oxide layer on an aluminum substrate.

Acid	Concentration. (M)	Voltage (volts)	Pore Size (nm)	Time (hours)	References
Oxalic	0.25	60	75	8,8	[40]
	0.3	40	Not specified	variable	[41]
	0.3	40	80	8, variable	[42]
	0.3	40	50	10,5 min	[43]
	0.3	60	80	3,8	[44]
	0.3	40	40–50	40 min, 2	[44]
	0.3	40, 50	20,35	variable	[45]
	0.3	30	40	8,10	[46]
	0.4	40	50	8,10	[46]
	0.5	50	80	8,10	[47]
	0.3	40	22	12,4,8,12& 16	[48]
Phosphoric	Not specified	195	200	variable	[9]
	0.4	5 to 40	20 to 75	1 step/variable	[34]
	0.4	80	80	1 step	[49]
	0.42	87 to 117	64 to 79	1 step/variable	[50]
Sulfuric	0.5	18	70	4, variable	[41]
	2.4	15 to 25	13 to 27	2 step/variable	[51]
	Not specified	12, 25, 40	25,50, 100	Not specified	[52]
	0.3	25	20	12, 4,8,12&16	[48]

##### 3.2.2.2. Non Acids and Neutral Solutions

The electrolyte composition largely determines whether the oxide layer formed is either barrier or porous. Barrier oxides are insoluble in the electrolyte, or dissolve at a much slower rate than it is deposited. In addition, the barrier layer tends to grow in non acid electrolytes and form an amorphous protective layer over the surface of the Al substrate. Commonly used non acid electrolytes are presented in Table 3.

**Table 3:** A selection of non acid electrolytes used to produce barrier layers.

Non Acid	Equation	Concentration (M)	pH	References
Ammonium Adipate	NH_4_OCO(CH_2_)_4_COONH_4_	150g/L	6.4	[29]
Sodium Borate	Na_2_B_4_O_7_	2.2	7	[20]
Sodium chromate	Na_2_CrO_4_	0.1	10	[38]
Sodium hydrogen phosphate	Na_2_HPO_4_	0.1	9.4	[38]
Sodium hydroxide	NaOH	0.01, 0.03 & 0.1	Not specified	[39]
Sodium sulfate	Na_2_SO_4_	0.1	5.8	[38]

#### 3.2.3. Voltage Conditions

In the electrochemical processing of Al to form an oxide layer, most studies have been made under the potentiostatic conditions of high voltage and low currents (refer to Figure 1). The voltages used are also dependent on the acid type and its molarity. Table 2 summarizes the voltage settings and times of the three main electrolytes used by several research groups in the manufacture of nano-porous oxide layer.

#### 3.2.4. Formation Mechanisms for Producing Barrier Layers and Nanoporous Alumina

##### 3.2.4.1. Introduction

The formation of a thick porous oxide layer on Al has been extensively exploited by industry for many years to provide an effective surface finish or as a pre-treatment for further surface processing, see Section 4.2.1. However, it has only being the recent advances in nanotechnology and the need to manufacture one dimensional materials that has initiated a considerable renewed effort into understanding the mechanism underlying the formation of the nano-porous membrane and its dependence on several macroscopic parameters. Although the anodization of Al had been known for many years, it was Keller *et al.* who undertook the first detailed investigation of the actual mechanism behind pore nucleation and the formation of the porous oxide layer [18]. As mentioned earlier, there are two types of oxide that can be produced; the first is the barrier layer or film and the second is the nano-porous oxide layer. In both cases the oxide formation process is heavily governed by the pH of the electrolyte composition involved and the operating conditions used.

##### 3.2.4.2. Barrier Layer Formation

The non-porous barrier oxide layer is insoluble or dissolves at a slower rate than it is formed in the electrolyte. Generally, the electrolyte used is neutral or a basic solution with a pH greater than 7 [53]. Typical solutions that have been used are ammonium borate, phosphate and tartrate compositions. In addition several organic acids such as citric, malic and glycolic have also been used, see Table 1. The barrier layer formed under these conditions is generally thin, non porous and rapidly forms under an applied voltage.

**Figure 4:**
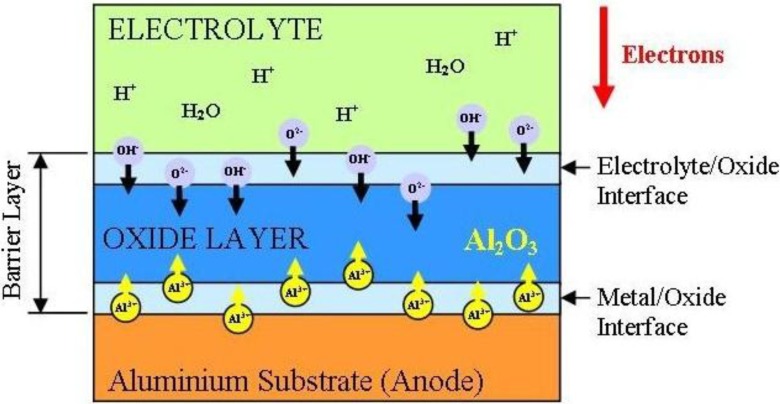
Schematic of the major features involved in the formation of the barrier layer.

The formation mechanism is straightforward and consists of two stages. In the first stage a constant current density is maintained, while the voltage increases linearly with time. To maintain the constant current density, the electric field strength must also be constant across the barrier layer, thus as the barrier layer grows the voltage increases until the formation voltage is reached. In the second stage the formation voltage is maintained while the barrier layer continues to grow by the migration of Al^3+^ ions outwards into the electrolyte and the inward motion of O^2-^ and OH^-^ ions [17], see Figure 4. The oxide formation occurs at both the metal/oxide and the oxide/electrolyte interfaces, with approximately 60% of the oxide growth occurring at the metal/oxide interface where the O^2-^ and OH^-^ ions combine with the Al metal. The balance of the porous growth occurs at the oxide/electrolyte interface where the Al^3+^ ions react with the water molecules in the electrolyte. As the thickness of the barrier layer increases, the electrical resistance also increases, the metal/oxide and oxide/electrolyte interfaces remain planar and the current flow decreases with time. Towards the end of this stage the barrier layer has fully formed and remains constant. Thus, the layer growth has decreased to a point where it is equal to the rate of dissolution. During this process the Faradaic current efficiency in building the oxide layer is high, close to 100% [54]. In addition, the resulting barrier layer thickness is directly proportional to the applied voltage and is approximately 1.0 to 1.4 nm per volt. The formation of the oxide tends to produce a level surface, with any initial minor surface imperfections such as roughness being filled-in by the forming oxide. The resulting oxide thickness is generally uniform and any surface flaws in the metal surface tend to produce localized current concentrations that result in some increased thickening of the oxide. At the end of this anodization process the surface chemistry is stabilized and resists any further reactions with its environment. In addition, the resultant barrier layer is hard, wear resistant and behaves as an electrical insulator.

##### 3.2.4.3. Nano-Porous Alumina Formation

The formation process of the nano-porous oxide layer is a complex process that produces a self organized hexagonal pore array, these hexagonal honeycomb structures have been reported by several researcher’s [4,55,56,57,58,59,60,61]. Unlike the barrier layer formation mentioned in the previous section, the porous structure consists of a thin non porous oxide layer of constant thickness that is adjacent to the metal substrate that continually regenerates [62] at the base of the pore while the pore wall is being created, this wall increases in height with time. The particular electrolyte, its concentration, the anodic voltage and bath temperature are the main parameters in determining the pore size and the distance between pores.

Typical electrolytes used to produce this type of oxide layer have a pH that is less than 5, and slowly dissolve the forming oxide layer. Examples of the acids used are sulfuric, phosphoric and oxalic. However; mixtures of organic and inorganic acids have also been used, see Table 1 and Table 2. The properties of the electrolyte are important in the formation of porosity and permeability. Electrolytes that are composed of less concentrated acids tend to produces oxide coatings that are harder, thicker, less porous and more wear resistant than those composed of higher concentrated acids. But the most important factor that must be considered when forming the porous oxide is the electrolyte’s ability to sustain a significant flow of Al^3+^ ions from the metal substrate into the electrolyte. There are two mechanisms that are responsible for the loss of Al^3+^ ions from the metal substrate. The first is by the direct expulsion of ions by the applied electrical field and the second is the dissolution of the forming oxide layer. In addition, if there are regions of high current flows when the electric field is applied, an increased dissolution rate can result (field assisted dissolution) [63].

The origin of pore nuclei and the exact mechanism of pore nucleation are still largely unknown. Several formation models have been proposed [63,64,65]; one model explains that pore nucleation results from an electric field assisted local chemical dissolution [66] at the electrolyte/oxide interface and oxide generation at the metal/oxide interface. This model explains that oxides formed using electrolytes with a pH greater than 5 produces a barrier layer in which the Al^3+^ ions lost from the metal substrate are retained in the oxide layer. This layer has uniform thickness and is stable. However, for electrolytes with a pH less than 5, there is a significant flow of Al^3+^ ions into the electrolyte and as a consequence there are regions where the formation of new oxide at the oxide/electrolyte interface is unstable. This regional instability produces variations in the applied electrical field; this in turn results in an increased dissolution rate [67,68]. This mechanism produces an underlying metal/oxide and oxide/electrolyte interfaces that consist of a large numbers of hemispherical depressions per cm^2^ that corresponds to the pore density. In these depressions the electrical field tends to be more concentrated due to the focusing effect of the hemispherical shape, hence the increased dissolution rate. [26] In contrast, the electrical field is fairly constant over the surface of the barrier layer formed in an electrolyte with a pH greater than 5, where the oxide thickness is uniform and stable. These hemispherical depressions form the foundations of the resulting pore structures. The location of these depressions is also influenced by the initial surface topography, surface imperfections such as impurities, pits, scratches, grain boundaries and surface treatments prior to anodization.

Recently Patermarakis *et al.* [69] proposed a pore nucleation model that results from the spontaneous recrystallization of the unstable rare lattice of oxide formed at the surface of the Al adjacent to metal/oxide interface to a more stable denser nano-crystalline oxide located in the oxide layer. The resulting recrystallization ruptures in the surface and produces regions of rarefied oxide between nanocrystallites (anhydrous/amorphous). It is in these regions that pore nuclei form. Earlier studies by Habazaki *et al*. indicated the potential for the enrichment of alloying elements, dopants and/or impurities in the Al substrate adjacent to the metal/oxide interface [70]. The enrichment layers were found to be about 1 to 5 nm thick immediately beneath the metal/oxide interface and were a consequence of the oxide growth. These enrichment layers may also be involved in the initiation of changes within the oxide layer that promote pore nucleation. In a recent investigation by Zaraska *et al*. the presence of alloying elements in an Al alloy (AA1050) not only slowed the rate of oxide growth but also influenced structural features such as porosity, barrier layer thickness, pore diameter and pore density of the forming oxide layer [23].

**Figure 5:**
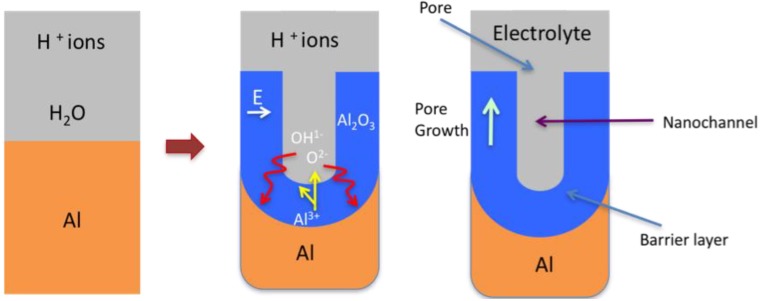
Schematic of the pore formation mechanism in an acidic electrolyte.

In the early stages of the anodization process Al^3+^ ions migrate from the metal across the metal/oxide interface into the forming oxide layer [71]. Meanwhile O^2-^ ions formed from water at the oxide/electrolyte interface travel into the oxide layer. During this stage approximately 70% of the Al^3+^ ions and the O^2-^ ions contribute to the formation of the barrier oxide layer [72], the remaining Al^3+^ ions are dissolved into the electrolyte. This condition has been shown to be the prerequisite for porous oxide growth, in which the Al-O bonds in the oxide lattice break to release Al^3+^ ions [73]. During the oxide formation the barrier layer constantly regenerates with further oxide growth and transforms into a semi-spherical oxide layer of constant thickness that forms the pore bottom, as shown in Figure 5.

During the formation of the porous oxide layer the anodic Al dissolution reaction is presented by

2Al → 2Al^3+^ + 6e^−^ (1)

and the resulting reaction at the cathode produces hydrogen gas:

6H^+^ + 6e^−^ → 3H_2_ (2)

Anode reactions taking place at the metal/oxide boundary (Oxygen anions react with Al)

2Al + 3O^2-^ → Al_2_O_3_ + 6e^−^ (3)

At the oxide/electrolyte boundary (Al cations react with the water molecules)

2Al^3+^ + 3H_2_O → Al_2_O_3_ + 6H^+^ (4)

Sum of the separate reactions at electrode (Overall anodization of Al equation)

2Al + 3H_2_O → Al_2_O_3_ + 3H_2_ (5)

**Figure 6:**
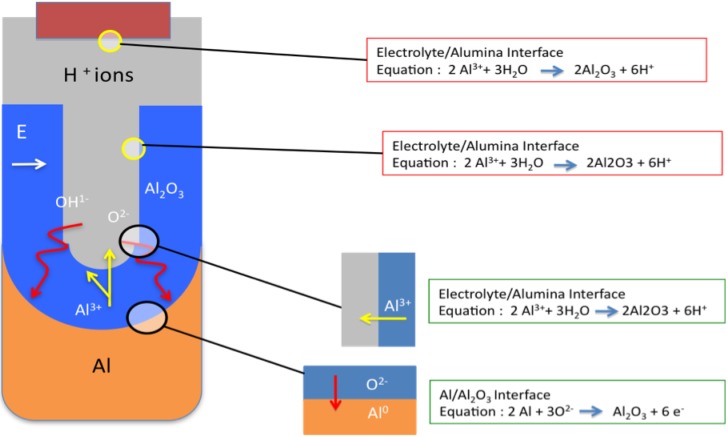
Schematic of ion movement during pore formation.

The steady state growth results from the balance between the field-enhanced oxide dissolution at the oxide/electrolyte interface at the base of the hemispherical shaped pores where the electric field is high enough to propel the Al^3+^ ions through the barrier layer and the oxide growth at the metal/oxide interface resulting from the migration of O^2−^ and OH^-^ ions into the pore base oxide layer, see Figure 6. This also explains the dependence of the size of the pore diameter to the electric field produced by the anodizing voltage. It should also be noted that the electric field strength in the pore walls is too small to make any significant contribution to the flow of ions.

The oxidation takes place over the entire pore base and the resulting oxide material grows perpendicular to the surface, neighboring pore growth prevents growth in any other direction. The vertical growth of the pore wall creates a columnar structure with a high aspect ratio that contains a central circular channel. This channel extends from the base of the pore to the surface of the oxide layer. This upward growth of the pore wall was recently investigated by Garcia-Vergara *et al.* in which a tungsten tracer was placed into an initial oxide layer formed by an initial anodization step [68]. During the next stage of anodization, the position of the tracer was monitored and found to travel from the metal/oxide interface of the barrier layer located at the base of the pore towards the growing wall structure. This flowing motion of the tracer was credited to the mechanical stresses being generated by the continued formation of new oxide within the pore base and the repulsive forces set up between neighboring pores during the growth of the wall structure. These forces resulted from the volume expansion (by a factor of 2) during the oxidation of Al to alumina. This volumetric expansion factor results from the difference in the density of Al in alumina (3.2 g cm^−2^) and that of metallic Al (2.7 g cm^−2^). This volumetric oxide expansion at the metal/oxide interface also contributes to the hemispherical shape of the pore base. However, under normal experimental conditions, the volumetric expansion factor is less than 2. This is due to the hydration reaction that occurs at the oxide/electrolyte interface which results in the dissolution and thinning of the oxide layer.

The theoretical volume expansion factor of the porous oxide layer is 1.6 when the formation current efficiency is 100%. However, the experimental values are less than the theoretical value; the experimental expansion factor can range from 0.8 to 1.6. This variation results from varying anodizing parameters such as a lower current efficiency [57]. The volumetric growth of the oxide layer is also dependent upon the type of electrolyte (this effect is more evident when using phosphoric or sulfuric acids), the electrolyte concentration and the electric field created by the anodizing voltage. Furthermore, a research investigation by Li *et al*. has shown that a volume expansion factor of 1.4 could be achieved under optimal anodizing parameters, independent of the electrolyte used [57]. The porous oxide layer thickness and hence pore height can grow to many times the height of the barrier layer. It is usually during this oxide growth that electrolyte anions can integrate into the forming porous structure near the oxide/electrolyte interface, while pure alumina is predominantly found in layers close to the metal/oxide interface. In the wall structure of the pore, the major constituents consist of alumina (amorphous Al_2_O_3_), anions from the electrolyte [74] which can be as much as 20% [59] depending upon the electrolyte and the formation conditions [75], small quantities of water and nano-crystallites. It is also possible for voids to form in the pore walls during the growth of the oxide layer. Possible causes of these voids range from oxygen evolution during oxide formation to localized defects in the barrier layer. These defects produce a condensation effect that involves cations and/or metal vacancies at the metal/oxide interface, which subsequently become detached, and form the void [76].

Many of the current research studies of the porous oxide layer formed by the anodization of Al have shown that this layer is amorphous [77,78,79,80]. However, much earlier research, summarized by Diggle *et al.* discussed the contradictory research results of the period [15]. For example, the non porous barrier layer was regarded as amorphous and anhydrous, while the porous layer had been found to be both amorphous and crystalline. In the case of the barrier layer, under normal anodization conditions the layer will be amorphous. However, studies by Uchi *et al*. have shown that with the right growth conditions it was possible to have amorphous or crystalline Al oxide being produced during anodization [20]. To form a crystalline oxide structure; an Al substrate was first immersed in boiling water to form a hydrous oxide layer [oxy-hydroxide with excess water (AlOOH.H_2_O)]. The substrate was then anodized in a neutral borate solution at high temperatures, during which Al^3+^ ions move from the metal substrate to the hydrous oxide interface, where they combine and transform the hydrous oxide to crystalline Al_2_O_3_.

**Figure 7:**
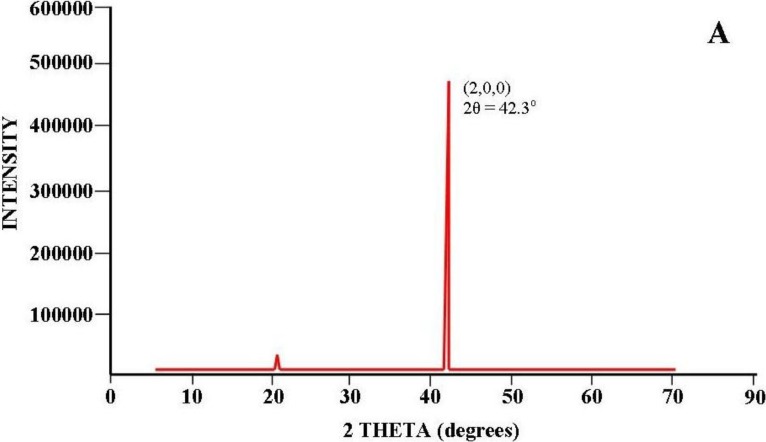

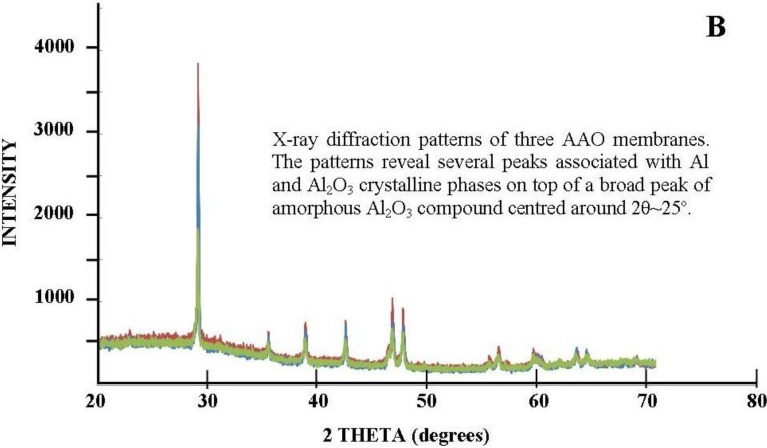
X-Ray Diffraction (XRD) Studies: Aluminum foil annealed under nitrogen at 500 °C for 5 hours. Anodic aluminum oxide (AAO) membranes prepared in a 0.3 M electrolyte of oxalic acid. XRD pattern of Al substrate prior to anodization (**A**) or after anodization (**B**) is shown.

Some of the contradictory evidence of the earlier works discussed by Diggle *et al.* [15] of the porous layer could be explained by the work of De Azevedo *et al.* [41]. In this study the structural characteristics of doped and un-doped porous Al oxide, anodized in oxalic acid was investigated using X-ray diffraction (XRD). The XRD patterns for the un-doped samples revealed several peaks associated with Al and Al_2_O_3_ crystalline phases on top of a broad peak that was approximately centered on the 2θ angle of 25°. This broad peak indicated that the synthesized layer was a highly disordered and/or amorphous Al oxide compound. The authors are currently investigating the structure of the porous layer of AAO membranes using XRD and can confirm the presence of peaks similar to those reported by De Azevedo *et al.* [41]. Figure 7 (a) presents the XRD pattern of an Al substrate prior to anodization; the crystalline form of Al is clearly shown. In Figure 7 (b) presents the XRD pattern of the Al substrate after being anodized in an electrolyte composed of 0.3 M oxalic acid. The XRD spectra were recorded using a Siemens D500 series diffractometer (Cu Kα = 1.5406 Å radiation source) operating at 40 kV and 30 mA. The diffraction patterns were collected at room temperature over a 2*θ* range from 20° to 70° with an incremental step size of 0.04°. The acquisition time was 2 seconds.

**Figure 8:**
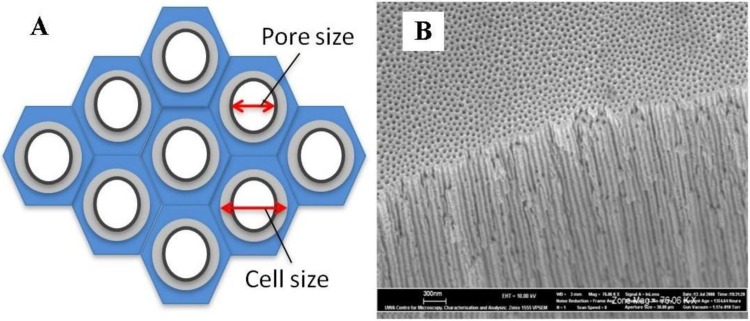
(**A**) Schematic of the ideal densely packed hexagonal array of pores; (**B**) Actual cross sectional view of a typical synthesized AAO membrane.

The mechanical stress generated at the metal/oxide interface during the formation of the oxide layer also influences the self-ordering of the developing pores. During the initial stage of anodization, the pores nucleate and develop randomly over the Al surface. As the anodizing time increases, the growth of the oxide layer progresses and characteristic pore patterns begin to immerge. The pore patterns continue to develop and become more ordered, with some self-adjustment of the pore configurations taking place [36]. Eventually, the number of various pore arrangements decrease until a single, long-range ordered pore structure is formed. The ideal structure consists of a densely packed hexagonal array of ordered pores of uniform size [81] see Figure 8. The pore density can range from 10^8^ to 10^12^ pores per cm^2^ depending upon the preparation parameters [44,52].

Characterization studies have revealed some interesting features of the pore structure; for example, the pore diameter remains uniform while the forming pore wall height increases linearly with time [82,83,84]. The same regular pore structure can be produced using oxalic, sulfuric and phosphoric acid solutions. Other acids that have been used are malonic and citric acids, which can also produce a regular pore arrangement under specific anodizing conditions [36]. The resulting in pore diameters can range in size from 4 to 250 nm [85,86] and the inter-pore distance can range from 50 to 420 nm [87,88]. In addition, the porous oxide structure, apart from being dependent upon the anodic voltage and time, is also dependent upon the electrolyte temperature, which has an important role in determining the thickness and mechanical properties of the oxide formed. For example; with constant voltage, temperature and concentration, membranes anodized in oxalic acid are found to have more uniform nano-channels, fewer embodied anions and superior hexagonal self-ordering than membranes anodized with sulphuric acid. In addition, membranes anodized in sulphuric acid were found to have lower flexibility, hardness and abrasion resistance [89]. At higher temperatures, generally above 60 °C, the porous oxide layer formed is thin, soft and non-protective, while at low temperatures typically between 0 to 5 °C, the resulting porous oxide layer formed is thick, compact and hard [90].

The effects of changing the anodizing parameters can directly affect the features of the pore structure [91]. For example, the electrolyte concentration can directly influence the size of the pore [46] and the cell diameter; while both pore diameter (D_p_) and inter pore distance (D_c_) are directly proportional to the anodizing voltage [92]. This relation was explained by Xu *et al.* in terms of the migration velocity of the reactive ions under the electric field during the growth and dissolution process of the forming porous oxide layer [85]. This explanation was later supported by the work of Shingubara *et al*. who investigated the voltage dependence in pore formation [9]. Furthermore, the linear correlation between pore diameter, cell diameter and voltage was established for citric, malonic, oxalic, phosphoric, sulfuric and tartaric acids by Ono *et al.* [36] The research to date has produced a set of equations that can predict both the D_p_ and D_c_ for porous anodic layers under potentiostatic conditions [23].

Pore Diameter:  D_p_ = λ_p_ U (6)

Inter Pore Distance Dc = λ_c_ U (7)

where λ_p_ is the pore proportionality constant = 0.9 nm per volt and λ_c_ is inter pore proportionality constant = 2.5 nm per volt. And since the pores are circular, the pore wall thickness W can be expressed by

Wall Thickness:  W = ½ (D_c_ − D_p_) (8)

The thickness of the barrier layer (B) at the base the pore remains constant during the stable potentiostatic anodization process and has been calculated empirically by Ebihara *et al.* for each acid [93]. The equations for the two commonly used anodization acids are presented below:

H_2_SO_4_ → B = 1.33 W (9)

H_2_C_2_O_4_ → B = 1.12 W (10)

Two other features that characterize the porous oxide layer are its porosity (α) and the pore density (n). For a densely compacted hexagonal pore array the porosity can be calculated from Equation (11) while the pore density for a surface area of 1µm^2^ can be calculated from Equation (12)

(11) $$\alpha =\frac{\pi }{2\sqrt{3}}{\left(\frac{D_{p}}{D_{c}}\right)}^{2}$$

(12) $$n=\frac{2.10^{6}}{\sqrt{3}D_{c}^{2}}=\frac{2.10^{6}}{\sqrt{3}U^{2}\lambda_{c}^{2}}$$

It should be noted that or a perfect self-organized hexagonal array of nano sized pores formed during optimal anodization conditions, the porosity should be 10% [94]. It can also be seen that the pore density is inversely proportional to the square of the anodization voltage. However, it should be pointed out that by increasing the anodizing voltage the electric field strength will increase. This generally improves pore ordering but there is a maximum voltage that can be attained before breakdown occurs. The breakdown voltage is dependent upon the electrolyte concentration, composition and temperature. During breakdown, other reactions such as solute oxidation, oxygen evolution and if there is a sufficiently large flow of electrons, sparks can even be produced. Generally, it can be seen that the lower the electrolyte concentration and temperature, the higher the breakdown voltage [53].

**Figure 9:**
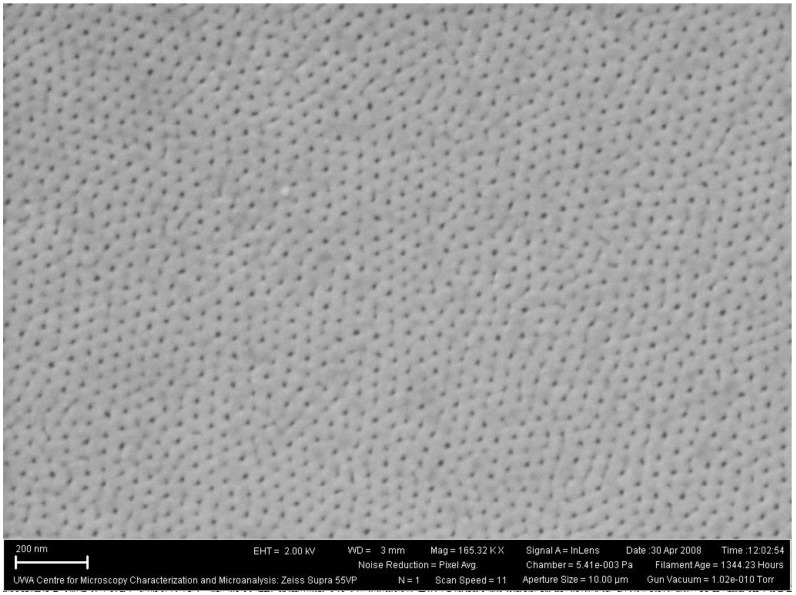
Scanning Electron Microscopy (SEM) image of self ordered pore array’s in an aluminum oxide layer.

The geometry of naturally self-organized ordering of cell arrays is far from the idealized model [94]. In this case, the pore and cell configuration usually does not display the highly ordered, densely packed, hexagonal columnar structure characterized by the ideal model. For example, Figure 9 presents a Scanning Electron Microscopy (SEM) image of a typical synthesized AAO membrane after a single short anodization step. The image reveals a self ordered structure that has no densely packed pore arrangement or any long range order in the oxide layer.

In addition, the naturally occurring self-ordering of the pores has also limited the size of both the pore and cell produced during anodization process. Studies have shown that the cell size is generally constrained to around 100 nm. However, AAO membranes used in optical devices, particularly in the infrared region of the spectrum require larger cell sizes. These larger cell sizes are possible by controlling the anodization parameters of electrolyte, electrolyte concentration, temperature, current density and anodizing voltage. For example, one procedure that is used to increase the pore/cell size uses phosphoric acid and a steady state voltage of 195 V, the result is a self ordered AAO membrane with cell sizes around 500 nm [4]. Further studies into the growth kinetics of porous layers formed by anodization in phosphoric acid under galvanostatic conditions have shown that the rate of growth of the oxide layer increases with increasing current density. At low current densities, (7.5 mA/cm^2^) the increase in the growth rate is proportional to the temperature. While at higher current densities, (17.5 mA/cm^2^) increasing the temperature has no effect on the growth rate. These studies have also revealed that the anodization time influenced the porosity of the oxide layer and that the layer growth and the oxide dissolution rate controlled the layers morphology [73,95].

##### 3.2.4.4. Two-Step Anodization of Aluminum

AAO membranes can be manufactured using a one, two or three-step anodization process. In the previous section the one step anodization process was discussed, during this discussion it became evident that the naturally occurring, self-organizing of the pore/cell array was far from an idealized model. The lack of a highly ordered pore/cell array results from the random nature of the initial formation mechanism. Investigations by Masuda and Fukuda in 1998 revealed that a highly ordered hexagonal structure could only be achieved under specific anodizing parameters and that the formation of the AAO membrane followed a self-organized growth mechanism [4]. During this study, they were able to demonstrate that the ideal pore array was characterized by an extended anodization period (up to a maximum of 160 hours). It was during this long anodization period that the pores were able to ‘self-adjust’ from their random initiation positions. The ordered pore positions are only observable at the metal/oxide interface after the barrier layer is removed. The pore position at the oxide/electrolyte interface reflects the initial random nucleation sites that were produced during the early stages of formation [59].

**Figure 10:**
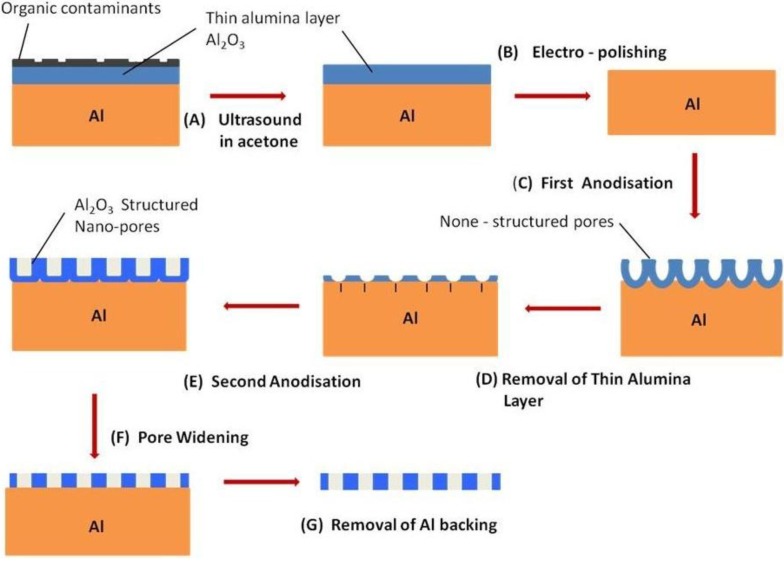
Schematic of the two-step anodization process.

To improve the pore/cell ordering on the oxide/electrolyte interface, a two-step anodization process was further developed by Masuda and Satoh [43]. The process permits the manufacture of a densely packed ideal hexagonal configuration of straight and parallel pore channels from the metal/oxide interface to the oxide/electrolyte interface. It was this macroscopically fabricated nanostructure membrane that spurred a renewed interest in using AAO membranes as possible templates for the manufacture of nano-structured materials and devices [36,46,96]. In the first step, a long anodization period is used to form a highly ordered pore array at the metal/oxide interface. The oxide layer is then removed to reveal a highly periodic and indented landscape covering the surface of the Al substrate. These indentations form the initiation sites for the formation of pores in the second anodization step [43,44,45,58,95]. And recently, self-assembled polystyrene spheres have been laid on the surface of the substrate to dictate the location of initiation sites during the first anodization stage [97]. During the second step, the pore/cell array formed is densely packed, highly ordered and the pore channels are straight and parallel [82,84,98]. Figure 10 presents a schematic of the two-step anodization process. It is this structure that has recently received intense interest for possible applications in the manufacture of nanomaterials [99]; some of these applications will be elaborated in Section 4. Further refinements in the anodization process have allowed Ho *et al.* to fabricate a multi-tiered three-dimensional nanostructure in the oxide layer [100]. Continuing research into controlling the position of the initial pore/cell sites has resulted in the development of several techniques. These techniques range from pre-texturing or nano-imprinting the surface using a mould or template, to ion beam lithography prior to the anodization process and hence control the ordering of the hexagonal pore arrays [101,102,103,104]. It should be noted that using techniques such as ion beam lithography requires the use of sophisticated and expensive equipment, unlike the two-step anodization process which uses relatively inexpensive laboratory equipment.

The major advantage in using the two-step anodization process is that the second stage of anodization procedure produces a superior porous oxide layer. This results from the oxide growth being dictated by the first step, whether it was by initial anodization followed by oxide removal or by a surface pre-treatment such as pre-texturing. It also means that there are no long periods of self adjustment required, so there is a high degree of pore/cell ordering achieved in a much shorter time frame. In addition, the current density profiles for the two-step anodization process have revealed that the first step requires a much longer time frame to achieve a consistent growth rate (steady state) in the porous oxide layer when compared to the second step. This confirms the advantage of using the two-step anodization process, since the removal of the oxide formed in the first step exposes the highly periodic and indented surface. This allows the fast initiation of pore nucleation and ordering to take place in the subsequent anodization step. As a result, the steady state growth of the oxide layer is achieved in a much shorter time frame [44], see Table 2 and Table 4. There have also been investigations into a three-step anodization process; in this process the oxide layers obtained after the first and second anodization steps were chemically removed. Then the Al substrate was anodized for a third and much longer time. This process did not show any significantly improvement in the ordering of the pore/cell array [105].

**Table 4:** Typical two-step Anodization Parameters for common porous forming electrolytes.

**Electrolyte**	**Concentration** (M)	**Potential** (V)	**1st Anodization** (Hours)	**2nd Anodization** (Hours)
Sulfuric Acid	0.3	24	5	3
Oxalic Acid	0.3	30	8	5
Oxalic Acid	0.3	60	5	3
Phosphoric Acid	2.5	60	8	5

Once the anodization process has been carried out it is necessary to remove the porous oxide layer from the Al substrate. Before this is done a thin layer of Acrifix 192 [106] or similar polymer is applied to the anodized side of the substrate. This technique reinforces the fragile AAO membrane and makes it easier to handle during the removal of the Al substrate. The substrate is removed using a solution of 0.1 M copper chloride in 7% hydrochloric acid. Once the substrate has been removed the barrier layer oxide is then etched in phosphoric acid, which also results in complete dissolution of the acrylic support and results in a clear film. An alternative technique that has been used to remove the Al substrate employs a one-step detachment mechanism. The procedure is carried out in a highly concentrated electrolyte, while a voltage, usually 5–10 V higher than the oxide forming potential is pulsed over a suitable time period resulting in the oxide layer being separated from the substrate [107].

After the separation of the oxide layer from the Al substrate, the diameter of the pores can be increased. Pore widening is done to selectively increase the pore diameter to a desired dimension. This adjustment is achieved by chemically etching the oxide surface within the columnar pore channel over a period of time. A commonly used etchant is phosphoric acid and typical times can range from 20 minutes to 85 minutes depending upon the voltage potential, concentration and temperature. The longer the dissolution rate the larger the pore diameter (D_p_) produced. It should also be mentioned that the thickness of the oxide layer is also reduced during pore widening. If the dissolution rate extends over an even longer period, the pore walls become so thin that they start to break up. In a recent investigation, Ye *et al.* was able to demonstrate the ability to control of the morphological transformation of the surface nano-structures of the oxide layer during the anodization process [108]. By increasing the anodization time and adjusting the current density, a longer dissolution period occurs, which effectively increased the number of broken pore walls. As the anodization process progressed, the broken pore walls began to emerge on the surface of the oxide layer. And as the time continued to increase, these damaged wall structures began to form bird nest like structures over the surface. These structures were interlaced with tens of thousands of nano sized sheets and wires. Under the conventional anodization process, the AAO surface is hydrophilic with a typical water contact angle of around 64°. However, the resulting bird nest structure effectively creates a super-hydrophilic surface with contact angles less than 10° [108].

##### 3.2.4.5. A Typical AAO Membrane Fabrication Technique

Al foil strips (various sizes) (99.99%) are annealed in nitrogen at 500 °C for 5 hours to recrystallize and release mechanical stresses from the samples. The Al strips are then degreased in acetone and etched in 3.0 M sodium hydroxide for 5 minutes before a thorough washing using milli-Q water. A layer of polymer is then applied onto the matte side of the strip. The strip is then anodized using a voltage of 60 V in an electrolyte solution of 0.3 M oxalic acid for 5 hours. The resulting thin layer of oxide is then exposed to a mixture of phosphoric and chromic acid (70 mL/L and 20 g/L, respectively) at 60 °C for 1 hour. This step is necessary to selectively remove the oxide layer and expose the textured substrate for the second anodization step. This procedure is schematically presented in Figure 10. The second anodization is performed under the same conditions as the first step, except the period is 3 hours. This gives rise to a regular, honeycomb arrangement of nano-pores, extending across the oxide layer. The nano-pores are then widened by chemical etching in 5% phosphoric acid at 35 °C for 15 minutes. A thin layer of Acrifix 192 is then applied onto the anodized side of the Al foil. This serves as a physical support for the AAO membrane during the removal of the Al substrate using an acidic solution mixture of 0.1 M copper chloride and 7% hydrochloric acid. Following this; the barrier layer oxide is then etched in phosphoric acid, complete dissolution of the barrier layer and acrylic support results in a clear membrane. The AAO membranes are then cleaned in a hot solution of 30% hydrogen peroxide for 15 minutes, dried and then stored in airtight containers ready for future applications.

## 4. Applications of AAO Layers and Membranes

### 4.1. Introduction

In this section we look at a variety of applications that use AAO layers and membranes. Firstly, we examine the more traditional engineering applications of the AAO layer in Section 4.2.1. We begin this section by examining the earliest application of the AAO layer, which was to form a protective barrier between the Al surface and the environment, thus preventing corrosion. Next we look at the incorporation of colored dyes into the AAO layer for decorative purposes and finally we examine the use of the surface texture to improve adhesion between glued components and as a surface pre-treatment prior to painting. In Section 4.2.2 we examine the electrical and photoluminescence properties of the AAO membrane. And finally, in Section 4.3 we examine the nanotechnology applications of AAO membranes in the biological and nano-biological fields.

### 4.2. Engineering Applications of AAO Layers

#### 4.2.1. Pore Sealing, Dyeing and Surface Textures

The porous oxide layers open pore structure provides poor corrosion resistance, the only resistance being provided by the impervious thin barrier layer adjacent to the Al surface. Historically, the most effective way to improve the corrosion resistance of Al components once they had been anodized was to seal the pore structure and form a thick protective barrier between the Al surface and the environment. The sealing process involves the immersion of the oxide layer in hot water. During this process the outer surface and the walls of the pores reacts to form a crystalline hydrate known as boehmite [109].

Al_2_O_3_ + H_2_O → 2AlO(OH) (13)

This inert, stable hydrated oxide fills the internal pores and provides a substantial protective layer over the entire surface.

Clear anodization uses sulphuric acid as the electrolyte, the most used anodization process, to produce a porous colorless oxide layer, typically between 1.5 µm to 25 µm. Oxalic acid, on the other hand, tends to produce a very pale yellow colored layer, which is much thicker. After anodization, colorization is achieved by immersing the Al components into a dye solution. The layer readily absorbs the dye solution, which normally takes several hours. The time depends on the dye solution properties, the dye solution temperature and the diffusion rate of the dye. Generally oxalic acids are dyed yellow or gold in color. A common problem that arises when using dyes is their poor fade resistance. This is dependent on the drying time, the dye concentration in the bath and the depth of penetration of the dye into the oxide layer. In addition, some organic dye colors are more prone to fading than others, while inorganic dyes tend to be more fade resistant. To seal the dye in the oxide layer, the Al component is immersed into a hot water bath, which closes the pores and traps the decorative dye within the pore structure [13]. A recently developed technique for coloring the oxide layer involves the deposition of metal or metal alloy pigments (*i.e*., Sn, Ni) into the pores. This process produces colored Al components that can resist fading over long periods of environmental exposure [17].

Excellent corrosion resistance can be achieved using the thick porous structure in conjunction with a suitable surface treatment and protective protocol. For example, in the aerospace industry Al is anodized in chromic acid, which produces an oxide layer with an effective thickness between 0.5 µm to 18 µm. The layer is difficult to dye, but provides an effective surface treatment prior to painting. It should be mentioned that because of the health risks associated with the use of chrome, this process is being phased out. In a similar aerospace application, anodization in phosphoric acid has been used as a surface pre-treatment before the application of adhesives used to glue components together.

In addition, the porous oxide layer formed on Al during the anodization process is amorphous, hard, has good corrosion resistance and is wear resistant. This makes the surface suitable for load bearing applications. Unfortunately the surface layer has a high coefficient of friction, which makes it unsuitable for many applications. To resolve this problem, investigations into enlarging the pore diameter and filling the pores with a suitable lubricating material were carried out by Tao *et al*. [110]. The study revealed that the lubricant (MoS_2_) produced in the pores of the oxide layer had little or no effect in reducing the frictional resistance. The study recommended a further investigation into developing a technique that could build up a solid lubricating film on the surface from stored lubricant in the pore structure.

#### 4.2.2. Electronic and Photoluminescence Properties of AAO Membranes

A major electronic application of the anodic oxide layer on Al uses the barrier layer to form a dielectric barrier in electrostatic capacitors. The construction of a typical capacitor consists of two Al foils. One foil is anodized to produce a barrier layer (this layer forms the dielectric layer in the capacitor) and is separated from the second Al foil by a spacer that is impregnated with an organic electrolyte. To improve the capacitance, an appropriate surface area magnification technique is used before the formation of the dielectric barrier. The increased surface area is achieved by first etching the Al foil and then sintering tantalum or niobium powder onto the surface, thus increasing the surface area [53]. Alternative techniques have been investigated, such as increasing surface roughness and enhancing the structural features of the barrier layer [20,29].

Photoluminescence only occurs in Al anodic oxide layers that are formed in organic acids such as citric, tartaric and oxalic. For example, oxide layers formed in low concentrations of oxalic acid are thick, exhibit a yellowish brown color, but when irradiated by ultra violet light they produce intense photoluminescence [32]. Similar investigations using AAO oxide layers that were doped with rare earth ions during the anodization process were also found to exhibit luminescence. This technique has been used to produce rare earth activated luminescent thin films for flat panel displays and optoelectronic devices [41].

### 4.3. Nanotechnology Applications

#### 4.3.1. Introduction

Nanotechnology research to a large extent is focused on the controlled fabrication of functional nano-scale structures and devices. Due to the small dimensions of these nano-elements, a bottom-up self-assembly process often provides a viable approach to overcome the current difficult technological challenges faced by researchers and engineers. One of the most important aspects of self-assembly lies in the capability of producing uniform structures over a large area using inexpensive chemical or biological processes. Al anodization is a relatively inexpensive electro-chemical and controllable process that allows the self-assembly process to dictate the development of porosity, mechanical properties and morphology of the AAO membrane. These membranes with their cylindrical pore geometry can be used as a template for the fabrication of nanostructures and nano-scale devices. For example, Masuda *et al*. has shown that AAO membranes with large pore sizes can be used in optical devices, particularly in the infra red region of the spectrum [4]. While Martin *et al.* has discussed the application of AAO membranes in molecular filters [111].

#### 4.3.2. Nanomaterials and Devices

##### 4.3.2.1. Nano-Dots and Nano-Magnets

The controlled placement of nano-dots on a substrate can be achieved with great accuracy using electron beam lithography. The major disadvantages of using this technique are the high capital cost of the equipment and the long exposure times which result in a low production output. An alternative process for the large scale fabrication of nano-dots on a semiconductor substrate uses an AAO membrane as an evaporation mask. The membrane provides an array of uniformly sized pores and site controlled pore locations where the nano-dots can be deposited. Before being used as a mask, the barrier layer is removed from the AAO membrane using a suitable etching technique, leaving a through pore structure or nano-channel. Masuda and Satoh [43] have used this technique to produce a highly ordered gold (Au), nano-dot array over a large area of silicon (Si) substrate [43]. Several other nano-dot arrays, such as Co, Fe and Ni have also been deposited on substrates such as Si and GaAs using this technique [112]. The major advantage of manufacturing nano-dot arrays using an AAO membrane is that the membrane can be used as a template. The template provides the precise location of individual pores in a high-density pore array without the need for expensive lithographic processes. The potential technological application of nano-dots is in the fabrication of ultra small electronic devices, ultrahigh-density recording media, and nano-catalysis applications [113].

Recently, the discovery of nano-magnets (also known as neodymium magnets or neomagnets) has made it possible to manufacture an economically competitive alternative to the SmCo and ferrite magnets that are currently in use. The nano-magnets display a greater magnetic strength for their size when compared to conventional magnets, which allows them to be made smaller and hence lighter for the same magnetic strength. The reduced weight of the nano-magnet makes them suitable for potential applications in head actuators for computer hard drives, magnetic resonance imaging (MRI) sensors, loudspeakers, headphones, magnetic bearings and couplings. In addition, these small powerful magnets have been used in bracelet clasps and in children’s magnetic toys [114].

##### 4.3.2.2. Nanowires/Nanorods/Nanotubes

Large arrays of one dimensional nanostructures, such as nanowires, nanorods and nanotubes, have attracted a great deal of interest in recent years, due to their potential applications in fields such as magnetic devices, electronics and optoelectronic devices. Fabricated AAO membranes with closely controlled nano-pore arrays have shown significant structural strength and have been used at high temperatures. It is these physical and mechanical properties that make the AAO membrane an attractive template for the manufacture of one dimensional nanocrystals such as nanowires, nanorods, nanotubes, and recently used as mask for x-ray projection lithography [115].

The advantages of using the AAO membrane as a template are firstly, that it allows the diameter of the nanowire, nanorod and nanotube to be tailored to the respective pore size in the membrane. Secondly, it ensures that the growth of the nanocrystal (nanowire, nanorod, nanotube) is aligned within the high aspect ratio nano-channel which is also perpendicular to the substrate surface at the base of the membrane. A wide variety of materials that include metals, oxides, conductive polymers and semiconductors can then be deposited into the pores of the membrane. Then a suitable formation mechanism can be used to generate nanowires, nanorods (short nanowires) and nanotubes. The dimensions of which can be controlled by adjusting the template pore geometry and the formation parameters [116].

**Figure 11:**
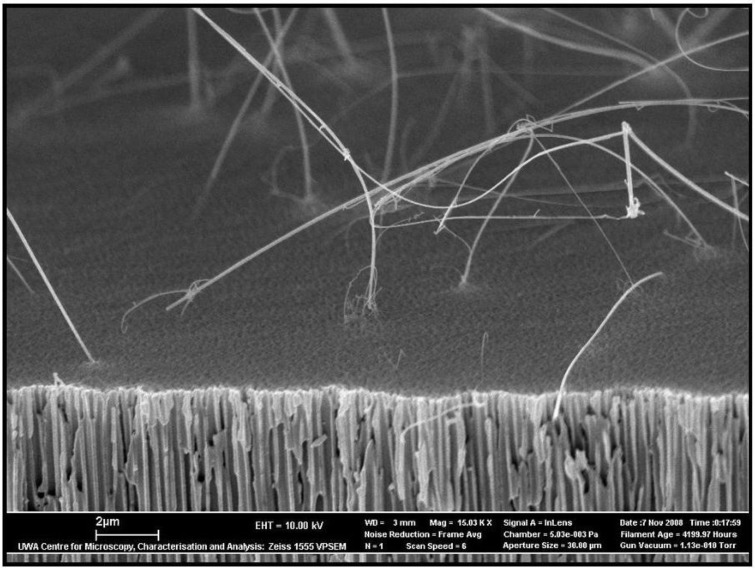
Cross-sectional images of an AAO membrane with silicon nanowires.

Recent studies have shown that nanowires/nanorods have the potential to make a significant contribution to the future development of new electronic devices and computer systems. For example, the semiconductor properties of nanowires composed of GaAs and GaP are known to produce significant rectifying characteristics [117]. This is the reason why a number of semiconductor devices such as junction diodes [118,119], transistors (FETs) [120,121], memory cells and switches [122], LEDs and inverter devices have already been manufactured using nanowire semiconductors [117]. As early as 1993, Hicks *et al.* were able to fabricate Bi_2_Te_3_ nanowires with the potential to be used in thermoelectric devices, since the nanowires displayed enhanced thermoelectric properties when compared to the bulk material [123]. In addition, recent studies using an AAO membrane template for a magnetic nanowire array had the potential to produce a recording density of about 155 Mbit/mm^2^. This would be a significant improvement on the currently available commercial hard drives that have a recording density of around 5.74 Mbit/mm^2^. This significant difference suggests that the AAO/magnetic nanowire device could be used in ultra-high density magnetic storage devices [124]. Furthermore, AAO membranes have also been investigated as potential templates for the manufacture of Si nanowires with novel properties (Figure 11). Other studies are investigating the magnetic properties of Co, Fe and Ni electrodeposited in AAO membrane templates [125].

AAO membranes that have incorporated a nanotubular structure within each pore of the pore array have recently received a considerable amount of interest due to their potential application in several newly emerging fields. These fields encompass a wide range of applications that include drug-delivery systems, molecular separation systems, single-DNA sensing devices, pollutant decomposition modules, hydrogen fuel devices, gas sensors and solar energy conversion devices [10]. The most attractive application of AAO membranes and nanotubes to date is their incorporation into a wide variety of sensors. One particular application of the membrane/nanotube technology is used in humidity sensors. These sensors exhibit high sensitivity, fast response and have a wide operational range when compared to other types of sensors [42]. The sensor has also gained acceptance as a reliable, low cost alternative for measuring relative humidity in industrial, meteorological and scientific applications. Furthermore, a combination of AAO membranes, Al composites and nanotubes has recently demonstrated their suitability for gas sensing detectors and a variety of similar sensors [126]. Similar studies by Chen *et al*. have shown that the AAO template could be used to direct and align the growth of α-Fe_2_O_3_ nanotubes within the pore channels. Once grown the composite structure could then be applied to sensing applications in lithium ion batteries and gas sensors [127]. In a similar application, Lee *et al.* were able to use carbon nanotubes (CNT) in field emitter tips [128]. Table 5 presents a selection of materials and processes currently being used to manufacture nanodots, nanowires, nanorods and nanotubes.

**Table 5:** Some materials and processes that are currently being used in the manufacture of nanodots, nanowires, nanorods and nanotubes.

Structure	Material	Substrate	Process	References
Nanodots	Mn	Si	Molecular beam epitaxy-scanning tunneling microscopy	[113]
			Ultrahigh vacuum System	
	Ag	Si	Molecular beam epitaxy	[129]
	InAs	Si	Electrochemical anodization	[130]
	SiO_2_, Ta_2_O_5_	Si, AAO	Electro-deposition	[131]
Nanowires	Au	Si	Electro-deposition	[132]
	Co, Fe	Au	Electro-deposition	[133]
	Ni, Bi	Al	Electro-deposition	[134]
	Au, Ag	AAO	Photochemical Synthesis	[135]
			Sol-Gel solution	
	LiMn_2_O_4_	AAO	Electro-deposition	[136]
	Fe/Co	AAO	Cyclic volt-ammetry	[137]
	Ag	Ag	Several Tested	[138]
	Au / Bi	Si, Pt,Cu, Ag,Pd	Electro-deposition	[139]
	Ferromagnetic	Au	Electro-deposition	[140]
	M-CdSe-M	AAO/Ti/Si	Electrochemical replication	[141]
	Ni	AAO/Ti/Si	Electro-deposition	[124]
	CdS	AAO/Au/Si	Electro-deposition	[142]
	Au	NW/AAO	Electro-deposition	[143]
	MnO_2_	AAO/Ti/Si	Electro-deposition	[144]
	M NWs	AAO, DMSO	Electro-deposition	[145]
	Bi_2_Te_3_	AAO	Electrochemical deposition	[146]
	Ag	Si	Ultrahigh Vacuum System	[129]
Nanorods	Al	AAO	Electro-deposition	[147]
	Ni	AAO / Al	Electro-deposition	[148]
	Pt	AAO	Magnetron Sputtering	[149]
	Pyroelectric triglycine sulfate	AAO	Aqueous Solution	[150]
	Au			
	Ni, Co, Au, Pb, Bi	Au	Cyclic volt-ammetry	[151]
	Polymeric (PMMA)	Au	Electro-deposition	[152]
Nanotubes	M-Al-O	AAO	Thermal decomposition	[153]
	ZnO,MgO, BaO		Calcination	
	C {C_2_H_2_}	AAO/Si	Plasma enhanced chemical vapor deposition (PECVD)	[154]
			
	C	AAO/Cr/Au	Chemical vapor deposition	[155]
	C	AAO/Fe/Ti/Al	PECVD	[156]
	C	AAO/Si	Thermal evaporation	[128]

#### 4.3.3. Biological Applications

The successful application of alumina as a biomaterial started in the early 1970s with the development of hip replacement technology [157]. Investigations since then have shown that osteoblasts can successfully adhere to and interact with the nanoporous structure of the AAO membrane, thus making the membrane suitable as a cell culture substrate [158]. The culturing of cells on both sides of the membrane seems to be a promising application in some fields of tissue engineering. A self-supporting AAO membrane can act as a physical barrier for the cultivation of different types of cells close to each other but without direct contact. The cells communicate via molecules, which diffuse into the pores and through the nano-channels to the other side of the membrane. There are two significant advantages in using AAO membranes for cell cultures. The first is being able to control the size of the pore diameter, which can be adjusted over a wide range. This in turn controls the diffusion of molecules through the membrane. And secondly, the membranes are optically transparent which enables the routine inspection of cellular growth and morphology using an optical microscope.

**Figure 12:**
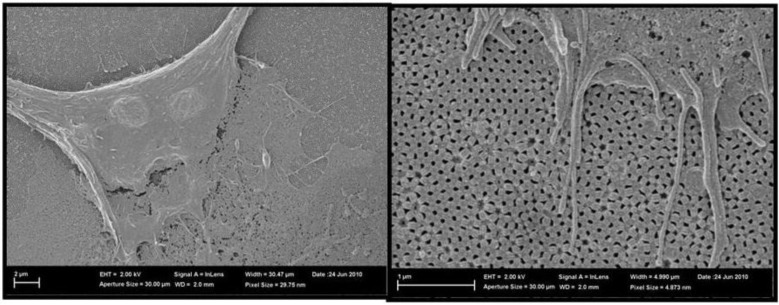
SEM Images of Madin-Darby Canine Kidney (MDCK) epithelial cells attached to an AAO membrane that was fabricated using Oxalic acid. (0.3 M Oxalic acid at 60 V for 5 h (1st anodization step) and 3 h (2nd anodization step); both steps carried out at 4 °C).

The results of Hoess *et al*. for cultivating liver cells (hepatoma cell line HepG2) on self-supporting and mechanically stable AAO membranes have demonstrated that the cells adhered to the membrane and proliferate during the culture period. The subsequent scanning electron microscopy (SEM) studies have revealed that the cells were homogenously distributed over the entire substrate with aggregations of larger cell clusters. The cells displayed normal morphology and developed microvilli for membranes with pore diameters greater than 230 nm [159]. In a recent study, the authors observed a similar cellular response of the Madin-Darby canine kidney (MDCK) epithelial cells on an AAO membrane with pore size of 80 nm. Figure 12 presents two SEM images of cellular attachment to the AAO membrane. The images clearly show the lamellipodia extending from the cell body, lying across the AAO surface and protruding into pore channels. In a similar investigation by Karlsson *et al.* using a primary human cell culture model on an AAO membrane revealed a normal osteoblastic growth pattern. The cell numbers steadily increased during the first two weeks. Maximum cell proliferation occurred on day 3, after which the cellular growth rate decreased. During this time the production of alkaline phosphatase (ALP) increased, thus indicating that the osteoblastic phenotype was being retained in the surface of the AAO membrane. In addition, cell adhesion was observed, with the cells having a flatten morphology with filipodia attached to the pores of the membrane. Both the SDS-PAGE and Western Blot measurements revealed that the membrane was able to absorb fibronetic. Furthermore, trace amounts of Al were measured in the surrounding medium, but no adverse effects were observed in the cell activity [160].

Natural bone consists mainly of inorganic nano-hydroxyapatite (HAP) (approximately 70%) embedded in an organic collagen matrix. HAP is critical to the bones durability, strength and its properties depend intimately on its nano-scale structure. Synthetically made nanophase HAP is recognized as an attractive material for bone tissue replacement therapies due to its compositional and biological similarity to native bone tissues. It is this bio-mimicry that allows the HAP to integrate with the surrounding tissues. AAO membranes have been successfully used as a template to control the growth of synthetic HAP [161]. In this procedure HAP precursors are deposited into the pores of the membrane, these precursors form the initial crystallization sites for subsequent deposits of HAP. The subsequent HAP deposits will then form a solid state structure that is structurally similar to the HAP found in natural bone [162]. The process of first depositing HAP precursors into the AAO template is similar to the mineralization process that occurs in natural bone, only in this case, the collagen matrix is the template [163]. Another advantage of the AAO membrane is that the surface roughness and the thick porous layer induce a positive response in bone growth. In similar studies of anodized titanium implants, this positive response of the bone tissues to the anodized surface has also been observed [28,164,165].

**Figure 13:**
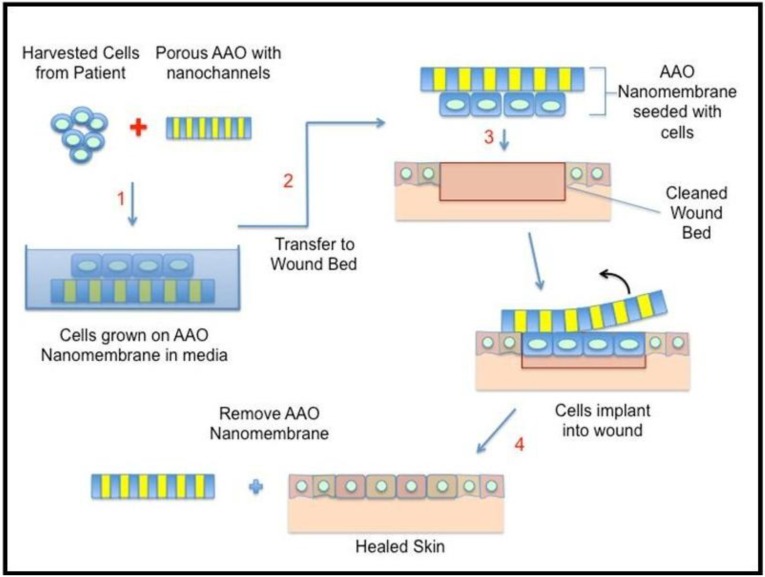
Schematic of AAO membrane used for skin tissue engineering.

Another application of AAO membranes in tissue engineering that is currently being investigated involves the manufacture of relatively large membranes that can be used in skin replacement procedures [166,167]. The function of these membranes is to allow skin cells to attach to the surface, encourage proliferation and assist in the formation of a confluent layer, see Figure 13. This cellular layer can then be lifted from the membrane and applied directly to the wound bed. Alternatively, the cellular layer and membrane can be applied to the wound bed, with membrane facing outwards. The advantage of the latter “band-aid” technique is that it membrane provides a protective barrier over the wound bed. The membrane allows the exchange of gases/fluids through the breathable nanochannels and at the same time prevents micron sized bacteria from entering the wound bed. Once the autologous cells have been incorporated into the wound bed, the outer AAO membrane can then be removed. There are two major advantages of this technology. The first advantage is that being totally inorganic the membrane can be sterilized more efficiently than a similar polymeric membrane. The second involves the membrane thickness, which ranges from 50 to 200 µm and its mechanical flexibility. Unlike the rigid alumina ceramics used in hard tissue engineering applications, the membrane can be easily shaped to conform to an individual patient skin or body shape.

A novel application of AAO membranes involves the membrane being formed into an immune-isolation macro-device, or capsule. This application has many appealing features for the encapsulation of cells or molecules within the body. The capsule membranes are extremely durable and important parameters such as pore size can be easily controlled during the manufacture of the membrane [168]. Studies have shown that by incorporating a poly (ethylene glycol) (PEG), coating to the capsule reduces the interaction between the serum albumin and the capsule contents. These capsules have also been examined for biocompatibility; *in vitro* tests have revealed that the capsule is non-toxic and implantation of these capsules into the abdominal cavity of rats induced only a minor transient inflammatory response [40].

Composite materials primarily composed of an AAO membrane can be used to provide an efficient immobilizing matrix for enzymes in biotechnology applications. Studies have also shown that these composites could also be used for sensor technologies and affinity separation. Composites composed of AAO membranes and poly (*ethyleneimime*) (PEI) or poly aniline (PANI) have also been investigated. Membranes composed of AAO and PANI have demonstrated good morphology and well developed porosity. While membranes composed of AAO and PEI have shown some flow related problems. Further work in the tailoring of these composites will enhance their properties and improve their performance [169].

AAO membranes have also been used as novel therapeutic platform structure for a hybrid-cell based sensing device that targets the *in situ* recording of cellular electrical activity variations resulting from changes in the surrounding micro-environments when therapeutic drugs are introduced. The device was developed by integrating both micro and nano fabrication techniques. The hybrid cell device uses the AAO membrane as the building platform, while the membranes capillary action and biocompatibility properties are incorporated into the device [53]. There are also micro-fluidic devices that are efficient diagnosis tools for detecting disease marker molecules. The construction of these devices consists of an AAO membrane being placed onto a glass substrate, which is then integrated within a poly (*dimethylsiloxane*) (PDMS) capsule. This composite structure forms a device containing large numbers of micro-channels. The pore size of the AAO membrane dictates the size of the channels and thus provides the versatility in selecting the size of the targeted proteins, molecules and concentrations. The PDMS micro-channel pattern was also found to assist in antibody immobilization and adhesiveness. The Hepatitis B surface antigen was used as the target protein during modeling investigations of the device. Modified versions of this device have been used as a sandwich-type Enzyme-Linked Immune-Sorbent Assay (ELISA). The performance of the modified device, when compared with a conventional ELISA system revealed a reduction in both the analytical volumes used and the times taken for analysis [170].

## 5. Concluding Remarks

Nanotechnology research to a large extent is focused on the controlled fabrication of functional nano-scale structures and devices. Al anodization is a relatively inexpensive electro-chemical and controllable process that allows the self-assembly process to dictate the development of porosity, mechanical properties and morphology of the AAO membrane. These membranes with their cylindrical pore geometry can be used as a template for the fabrication of nanostructures and nano-scale devices. In this article we have presented the electro-chemical process that produces the porous AAO membrane and a selection of applications where these membranes are currently being used or investigated for potential future application. The current applications clearly demonstrate the versatility of the AAO membrane and it is only with further research that their full potential will be discovered and new applications found.
